# Stochastic Channel-Based Federated Learning With Neural Network Pruning for Medical Data Privacy Preservation: Model Development and Experimental Validation

**DOI:** 10.2196/17265

**Published:** 2020-12-22

**Authors:** Rulin Shao, Hongyu He, Ziwei Chen, Hui Liu, Dianbo Liu

**Affiliations:** 1 Department of Mathematics and Statistics Xi'an Jiaotong University Xi'an China; 2 Department of Electrical Engineering Xi'an Jiaotong University Xi'an China; 3 Beijing Jiaotong University Beijing China; 4 Department of Mathematics Mianyang Vocational College Mianyang China; 5 Computer Science and Artificial Intelligence Laboratory Massachusetts Institute of Technology Cambridge, MA United States

**Keywords:** federated learning, differential privacy preserving, neural network pruning, health care, privacy, medical data, machine learning, neural network

## Abstract

**Background:**

Artificial neural networks have achieved unprecedented success in the medical domain. This success depends on the availability of massive and representative datasets. However, data collection is often prevented by privacy concerns, and people want to take control over their sensitive information during both the training and using processes.

**Objective:**

To address security and privacy issues, we propose a privacy-preserving method for the analysis of distributed medical data. The proposed method, termed stochastic channel-based federated learning (SCBFL), enables participants to train a high-performance model cooperatively and in a distributed manner without sharing their inputs.

**Methods:**

We designed, implemented, and evaluated a channel-based update algorithm for a central server in a distributed system. The update algorithm will select the channels with regard to the most active features in a training loop, and then upload them as learned information from local datasets. A pruning process, which serves as a model accelerator, was further applied to the algorithm based on the validation set.

**Results:**

We constructed a distributed system consisting of 5 clients and 1 server. Our trials showed that the SCBFL method can achieve an area under the receiver operating characteristic curve (AUC-ROC) of 0.9776 and an area under the precision-recall curve (AUC-PR) of 0.9695 with only 10% of channels shared with the server. Compared with the federated averaging algorithm, the proposed SCBFL method achieved a 0.05388 higher AUC-ROC and 0.09695 higher AUC-PR. In addition, our experiment showed that 57% of the time is saved by the pruning process with only a reduction of 0.0047 in AUC-ROC performance and a reduction of 0.0068 in AUC-PR performance.

**Conclusions:**

In this experiment, our model demonstrated better performance and a higher saturating speed than the federated averaging method, which reveals all of the parameters of local models to the server. The saturation rate of performance could be promoted by introducing a pruning process and further improvement could be achieved by tuning the pruning rate.

## Introduction

### Medical Data Privacy

Medical data analysis in health care brings many benefits and holds great promise for transforming the field. With the help of a wide range of health care networks, health care organizations are now able to analyze a vast volume of data with great variety and velocity to support decision making [1-3]. In addition, automated machine-learning algorithms could facilitate patients and physicians to make better informed choices by providing empirical estimates based on gigabytes of data [4]. Apart from decision support, medical data analysis could also promote analytical capability for patterns of use, analysis of unstructured data, predictive capability, and traceability [3].

However, health care data security and privacy issues have raised broad ethical and legal concerns in recent years given the sensitive nature of health information [5]. Health care research often involves studies of a large amount of data collected from various sources such as pharmacies, insurance companies, government agencies, and research institutions. For instance, to discover new drugs or assess a new therapy, a research institute may need clinical records provided by hospitals’ autonomous databases [6]. This direct sharing of medical data is likely to violate individual privacy and expose data owners to the threat of illegal data collection [7].

To address this data privacy concern, different countries have enacted different legislations and policies [8,9], which impose limitations on data collection and utilization for health care research. Over the years, many traditional methods for privacy preserving have been proposed, such as deidentification [10,11], a hybrid execution model [12], and identity-based anonymization [13]. However, as pointed out by several authors, these methods alone could not guarantee the anonymity and security of medical data [14-16]. Recently developed machine-learning methods require considerable data to acquire models with sufficient accuracy [17]. To leverage massive and diverse datasets and promote machine-learning models, the issue of balancing privacy and regulatory requirements has to be addressed [18].

### Federated Learning

In conventional deep learning, all training data are shared with a central server that performs the analysis. Having no control over this process, the clients that contribute the data may have to upload their sensitive information to the server without a guarantee of its security of privacy. Furthermore, the learned model is generally not directly available to the client so that they have to reveal the inputs to the cloud when using the model [19], risking privacy leakage in both the training and using processes. Federated learning can address this problem by introducing some algorithmic techniques that distribute the learning process to local devices so that the clients could keep their data private and obtain a local model for future use.

Federated optimization has been studied by Konečný et al [20,21] for distributed optimization in machine learning. This work introduced a setting for distributed optimization in which none of the distinctive assumptions [21] is satisfied, making federated learning a feasible alternative to other methods. The proposed framework is different from conventional distributed machine learning [22-27] owing to the huge number of clients, extremely unbalanced/nonindependent and identically distributed data obtainable for each client, and poor network connections [28]. To address the latter constraint, Konečný et al [28] proposed two approaches to reduce the uplink communication costs: structured updates and sketched updates. McMahan et al [29,30] advocated for federated stochastic gradient descent (SGD) and federated averaging algorithms as feasible approaches for the federated learning of neural networks based on iterative model averaging. As an alternative to protecting a single data point’s contribution to a learning a model [31], Geyer et al [32] proposed an algorithm for client-sided federated optimization to hide the specific contributions of individual clients during training. Further, methods to strengthen the reliability of federated learning, such as secure aggregation [33], essentially need synchronization on a rigid set of devices so that only a simple summation of the updates from users is consumed by the server side of the algorithm [34]. Applications based on federated learning algorithms have been proposed in several domains, ranging from content suggestions [35] to next-word prediction [36]. Bagdasaryan et al [37] focused on the vulnerability of federated learning. This work showed that the federated learning algorithm is vulnerable to a model-poisoning attack, which is different from poisoning attacks that target only the training data.

Besides the direct leakage of privacy mentioned above, participants in the distributed system may indirectly reveal some information about sensitive data via the weights uploaded to the server in the training process.

To address both direct and indirect privacy leakage of health care data, we developed the stochastic channel-based federated learning (SCBFL) method, which enables local participants to manipulate their data confidentially while benefitting the model’s performance from the server with only a small proportion of the locally trained gradients revealed stochastically to the central model.

## Methods

### Principle of the SCBFL Approach

**Figure 1:**
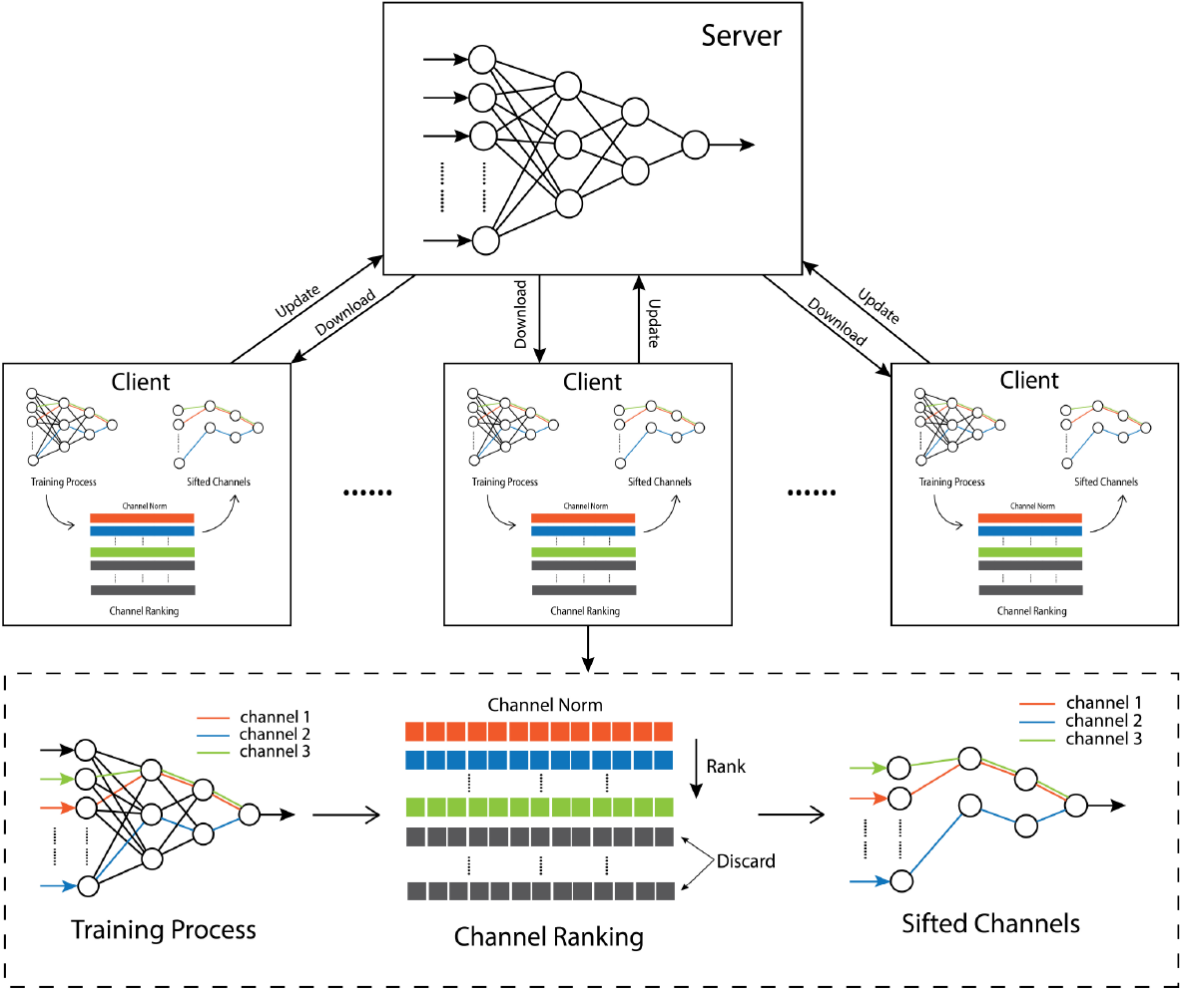
Schematic of the stochastic channel-based federated learning (SCBF) model.

Based on the observation that different features do not contribute equally to the training process and that the importance of each feature may vary from one dataset to another, SCBFL (Figure 1) was developed as a privacy-preserving approach that seizes the most vital information from the local training results only by uploading a small fraction of gradients stochastically. The intuition behind this method is that the biological neural circuit follows the law of use and disuse, and the strongest neurons for an individual are those that constitute an active circuit in the learning process, suggesting that the neurons in one artificial neural network are not independent throughout a specific training process. Thus, we could consider the collaborative effect of neurons in each channel (similar to a biological neural circuit) when selecting parameters for a server update: if a channel of neurons changes substantially in a training loop, we can assume that it is a strong neural circuit in the network, corresponding to a sensitive feature in the input sets, whereas the neural channels showing little change in one training loop should be regarded as deteriorated channels, whose information could be kept private with minimal effect on the server’s final performance. Choosing the channels with the most substantial variation enables SCBFL to only upload a small percentage of the gradients in each training loop while achieving comparable accuracy to the federated averaging method without uploading the integrate local weights to the server, as will be demonstrated in the Results section.

The update algorithm plays an essential role in SCBFL. In each global loop, SCBFL computes the norms of channels in gradients that result from the local training process, calculates the α-percentile of the norms, and then sifts out the channels with greater variation in the gradients than the percentile, in which α is the update rate set by the local participant. The sifted parameters are then used for the server update.

To facilitate the description of the algorithm, suppose there are *N* features as input and an *L*-layer deep neural network is conducted with *m_1_*, *m_2_*,…, *m_L_* neurons in each layer. For convenience, we denote *m_0_*=*N* as the input dimension, and we denote the weight matrix as *W*=[*W_1_*, *W_2_*,…*W_L_*] and the bias matrix as *B*=[*B_1_*, *B_2_*,...*B_L_*]. The shapes of the weight matrix and bias matrix could be expressed as follows:

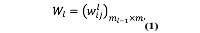

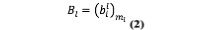





Where *l*=1,2,…*L*, and *m_l_* is the number of neurons in the *l*th layer.

The update algorithm includes five steps:

1. Train the local model: The local models are trained separately on their own datasets, and each model results in a gradient matrix showing the change in the weight matrix during each training loop. The gradient matrix *G* has the same shape as the weight matrix *W*. Since the influence from the bias matrix is negligible compared to that of the weight matrix, the changes in bias are omitted for the sake of efficiency.

2. Compute channel norms: Considering that a channel must go through a neuron in each layer and correlates to an *L*-dimensional vector comprising the index of these neurons, the results of a channel’s norm could be saved in an *L*-dimensional tensor *T,* each element of which equals a channel norm. The shape of *T* should be:

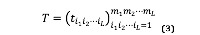



In addition, 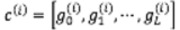 is the *i*th channel, in which 
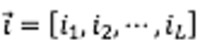
 is the index of the tensor that correlates the neurons this channel goes through in each layer. The Euclidean norm of each channel is calculated by

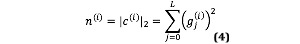



and is saved in the *L*-dimensional tensor *T*:

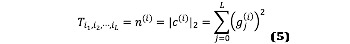



3. Sort norms: Given a fixed upload rate α (also referred to as “update rate” in this paper), we could straighten the gradient tensor to a vector and sort it, computing the α-quantile *q_α_* as a threshold for the channel selection.

4. Process gradients: There are two ways to process the gradients. With negative selection, the channels with norms below the α-quantile are discarded and the remaining parameters are selected for the update. With positive selection, the channels with a norm above *q_α_* are selected and the remaining parameters are set to zeros.

In our preliminary trials, both selection methods worked well. Considering that different neural channels may include the same neurons, positive selection tends to behave better than negative selection due to the preference to upload more parameters with the same update rate. Taking positive selection as an example, for each element *T_t1_, _t2_,*…,*_tL_* in tensor *T*, which corresponds to a specific channel, the gradients are processed with respect to the rank of this channel’s norm, as shown in the following form:

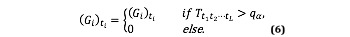



**Figure 2:**
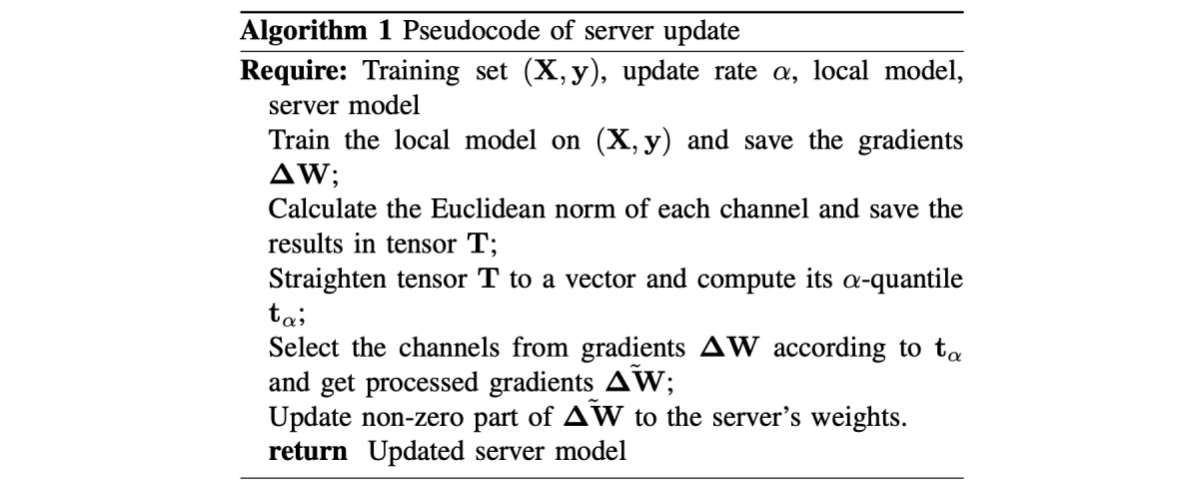
Pseudocode of the server update.

5. Update server: Finally, the processed gradient matrix G̃ is uploaded to the server, and the server will update its parameters by adding gradients G̃ to its original weights (Figure 2).

The server update algorithm is executed every global loop, and our experiment showed that with only 10% of local channels revealed, the server could have comparative performances to those of the federated averaging methods with higher speed to reach saturation. Before the next training loop begins, the local model downloads the server’s latest weights. The download rate was set to 100% since we suppose that the server weights could be shared publicly, which could be adjusted according to the application scenarios.

### Pruning Process

Training a model with privacy-preserving methods could be time-consuming, especially when the training sets are enormous. To address this problem, we introduced a neural network pruning process to SCBFL that could prune off the redundant nodes in the neural network based on the validation set, thus saving a substantial amount of time. This work is done circularly in the first several global loops until the distributed system reaches a suitable scale, so that SCBFL with pruning (SCBFLwP) learns from the datasets more efficiently.

**Figure 3:**
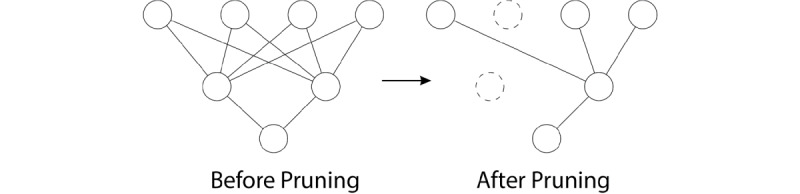
Neural network pruning.

Neural network pruning (Figure 3) is not a novel concept. Yang [38] proposed a method to prune connections based on the magnitude of weights. He et al [39] used a channel-pruning method to accelerate a deep convolutional neural network. Han et al [40] introduced a growing-and-pruning approach for a fuzzy neural network. Moreover, Srinivas [41] proposed a systematic method to prune one neuron at a time, addressing the problem of pruning parameters in a trained neural network model.

Given the fact that each neural network has a computation process consisting of multiplication, addition, and activation, neurons whose output consists mostly of zeros may have little effect on the output of subsequent layers, not to mention the final results [31]. Removing these redundant nodes from the model will do little harm to the accuracy of the network but will save abundant execution time.

Average percentage of zeros (APoZ) [42], which measures the percentage of zeros in the activations of a neuron under rectified linear unit (ReLU) mapping, is used to evaluate the redundancy of neurons in the network. 
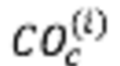
 is denoted as the output of the *c*th neuron in the *i*th layer. Let *M* denote the output dimension and *N* denote the total quantity of validation examples. 
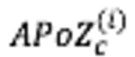
 of the *c*th neuron in the *i*th layer is then defined as:

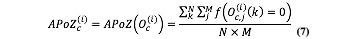



where f(·)=1 if true and f(·)=0 if false.

**Figure 4:**
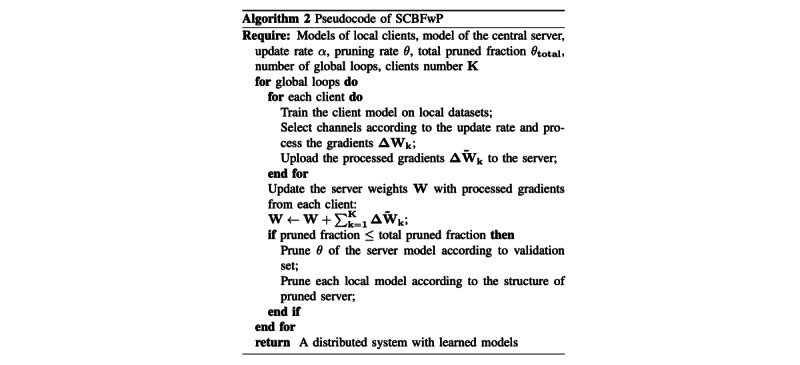
Pseudocode of stochastic channel-based federated learning with pruning (SCBFwP).

SCBFLwP (Figure 4) then decides which neurons will be pruned according to APoZ using validation sets: those having the highest APoZ will be pruned, the number of which is a fixed percentage of the total number of neurons left in each global loop.

### Distributed Learning Setting

We propose a privacy-preserving federated learning method based on the neural network. Federated learning could be executed on a distributed system such as a mobile device to achieve collaborative deep-learning goals with little risk of privacy leaks. Each device trains its model on the local dataset for several epochs in each global loop and only stochastically uploads a small percentage of the model weights to the server to achieve good performance in the server without sharing the local data or the overall model weights.

In our trial, we implemented a distributed system with 5 clients contributing to one server. Preliminary experiments were conducted to determine the proper structure for the proposed model. Through manual tuning, we found that the model achieves the best performance with high efficiency using 3 layers. Therefore, for each local client, we constructed an artificial neural network for binary prediction of mortality with 3 fully connected layers including 64, 32, and 1 neuron in the corresponding layers using ReLU activation at hidden layers and sigmoid activation at the output layer. We also added a dropout layer between the second and third hidden layers to reduce overfitting.

Regarding the parameters of communication between server and clients, the download rate was set to 100% for each client model, supposing the parameters of the server are shared publicly. The update rate was set to 30% for both the channel-based federated learning method and distributed selective SGD method. To enhance the influence of the latest update parameters, we chose 0.8 as the decay rate. As for the training process, we trained each model for 100 global loops and 5 epochs in each loop with the batch size set to 32.

We used the SGD algorithm to optimize our neural networks. Concerning the configuration of SGD, the learning rate is a hyperparameter that controls how much to adjust the model in response to the estimated error each time when the model weights are updated. Our experiments on testing models with various learning rates suggested that the proper learning rate was around 0.01 to guarantee both good performance and stable results.

In addition to the configuration of the model, the importance of performance measurement has long been recognized. With respect to the assessment of a classification model, area under the receiver operating characteristic curve (AUC-ROC) and area under the precision recall curve (AUC-PR) are reliable metrics: the higher the AUC value, the better the model is at distinguishing between patients in terms of mortality and survival.

### Dataset for the Experiment

The data used in our experiment were provided by hospitals, comprising 30,760 admissions with status information represented by alive or dead. To explore the relationship between mortality and admissions, we developed a model that takes the medications as inputs and predictions of binary mortality as the output. The cohort was managed for 2917 different medications in total. Information on whether a patient took each of the medications after admission was adopted as a binary input feature. We used 60% of the dataset for training, 10% as the validation set, and 30% as the test set. The training set was equally divided into five parts as local training sets.

### Statistical Analysis

The performances of models were evaluated by the AUC-ROC and AUC-PR, which are both typically used for measuring the performance of a classifier. To better understand the ROC curve, the concept of a confusion matrix first needs to be introduced.

A confusion matrix is a table consisting of four different combinations of prediction and ground truth, which are the true positive (TP), false positive (FP), false negative (FN), and true negative (TN). TP means that the model predicts the sample as positive and it is in fact positive. The value of TP can be calculated by counting the number of correct positive predictions. The other three parameters can be interpreted in a similar manner. With the help of a confusion matrix, more performance indicators can be defined, including the true positive rate (TPR), also known as recall and sensitivity, the false positive rate (FPR), and precision, which are calculated as follows:

TPR=TP/TP+FN **(8)**

FPR=FP/TN+FP **(9)**

Precision=TP/TP+FP **(10)**

The ROC curve is plotted with TPR against FPR at various classification thresholds, where TPR is on the y-axis and FPR is on the x-axis. Lowering the classification threshold means that the model will predict more samples as positive, thus increasing both FPR and TPR. As an alternative to ROC, the PR curve is plotted with precision against recall (TPR) at various classification thresholds. When datasets are imbalanced or skewed, the PR curve is a preferred alternative to the ROC curve. Both curves provide a visualization of model performance at different thresholds, and the AUC measures the entire two-dimensional area under these curves, providing an aggregate measurement of performance across all possible thresholds. Ranging in value from 0 to 1, AUC-ROC and AUC-PR can be interpreted as the possibility that the model ranks a positive sample more highly than a negative sample.

## Results

### Performance of the SCBFL Model

**Figure 5:**
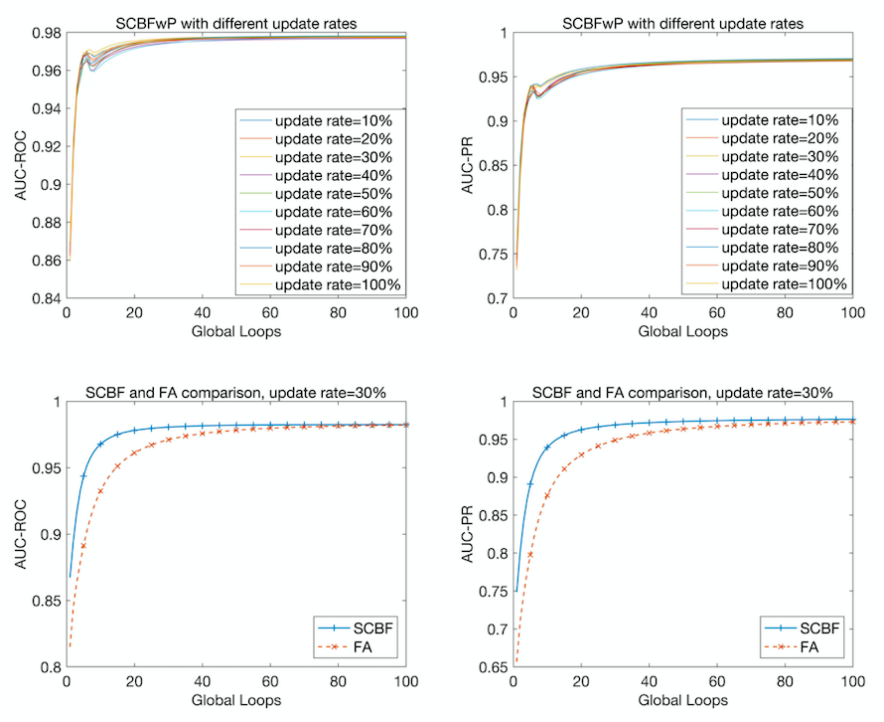
Performances of stochastic channel-based federated learning (SCBF) models. The top two graphs show the performances of SCBF with pruning (SCBFwP) using different update rates, and the bottom two graphs show the comparison between the performances of SCBF and federated averaging (FA). The left column shows the area under the receiving operating characteristic curve (AUC-ROC) and the right column shows the area under the precision-recall curve (AUC-PR) as performance metrics.

The update rate controls the number of selected channels whose nonzero part is uploaded to the server in each global loop, playing a vital role in affecting the final performance. To choose a suitable update rate for our distributed system, we implemented SCBFL models with different update rates ranging from 10% to 100%. Neural network pruning was used in this step to accelerate the training process. The performances are plotted in the first row of Figure 5. The result showed that even with 10% of the channels uploaded to the server, the SCBFL model achieved an AUC-ROC of 0.9776 and an AUC-PR of 0.9695, which outperformed the model that shared all of the parameters with the server. In addition, using a wide range of upload rates only led to a 0.01319 amplitude change in AUC-ROC and a 0.02739 amplitude change in AUC-PR, which facilitated the configuration process with stably high performance.

Table 1 compares the effectiveness of the SCBFL method with that of federated averaging, which is widely used in distributed systems and implements the federated learning by averaging the gradients obtained from local training processes [36]. For this comparison, we set the update rate to 30% for SCBFL and conducted both methods for federated learning on the same datasets for 100 global loops without pruning. As shown in Figure 5, our model reached saturation at the 20th global loop, which was faster than that obtained with federated average, which reached saturation at the 60th global loop. The performance of SCBFL consistently exceeded that of federal averaging. In the 4th global loop, SCBFL achieved a 0.05388 higher AUC-ROC and 0.09695 higher AUC-PR than those of federated averaging. After 100 global loops, the AUC-ROC and AUC-PR of SCBFL was 0.0033 and 0.0032 higher than that of federated averaging, respectively.

**Table 1:** Saturated performances of stochastic channel-based federated learning compared with federated averaging.

Method	AUC-ROC^a^	AUC-PR^b^
SCBFL^c^	0.9825	0.9763
Federated averaging	0.9821	0.9731

^a^AUC-ROC: area under the receiver operating characteristic curve.

^b^AUC-PR: area under the precision-recall curve.

^c^SCBFL: stochastic channel-based federated learning.

**Figure 6:**
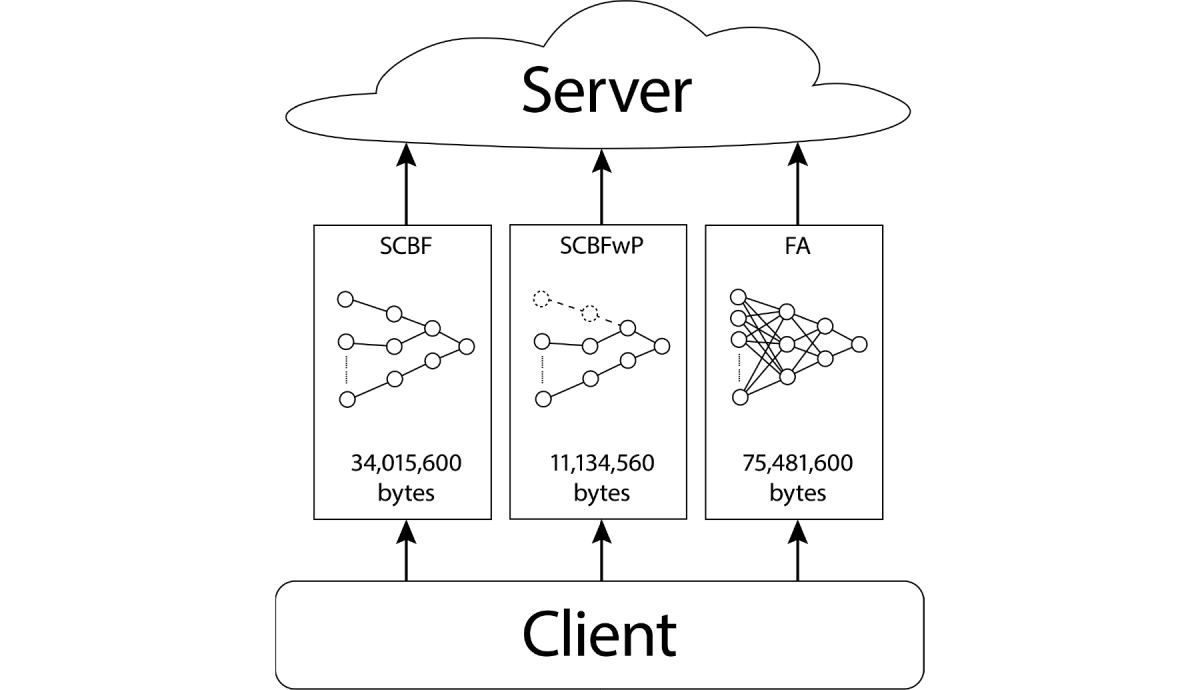
Trans-information for upload processes using different methods. The stochastic channel-based federated learning with pruning (SCBFwP) method could save 85% of the trans-information compared to federated averaging (FA), and stochastic channel-based federated learning (SCBF) could save 55% compared with FA.

As shown in Figure 6, when the upload rate for the channels was set to 30%, 45% of the parameters were uploaded to the server using positive selection. With half of the parameters unrevealed to the server, the model achieved better performance and higher saturating speed.

### Performance of SCBFLwP

**Figure 7:**
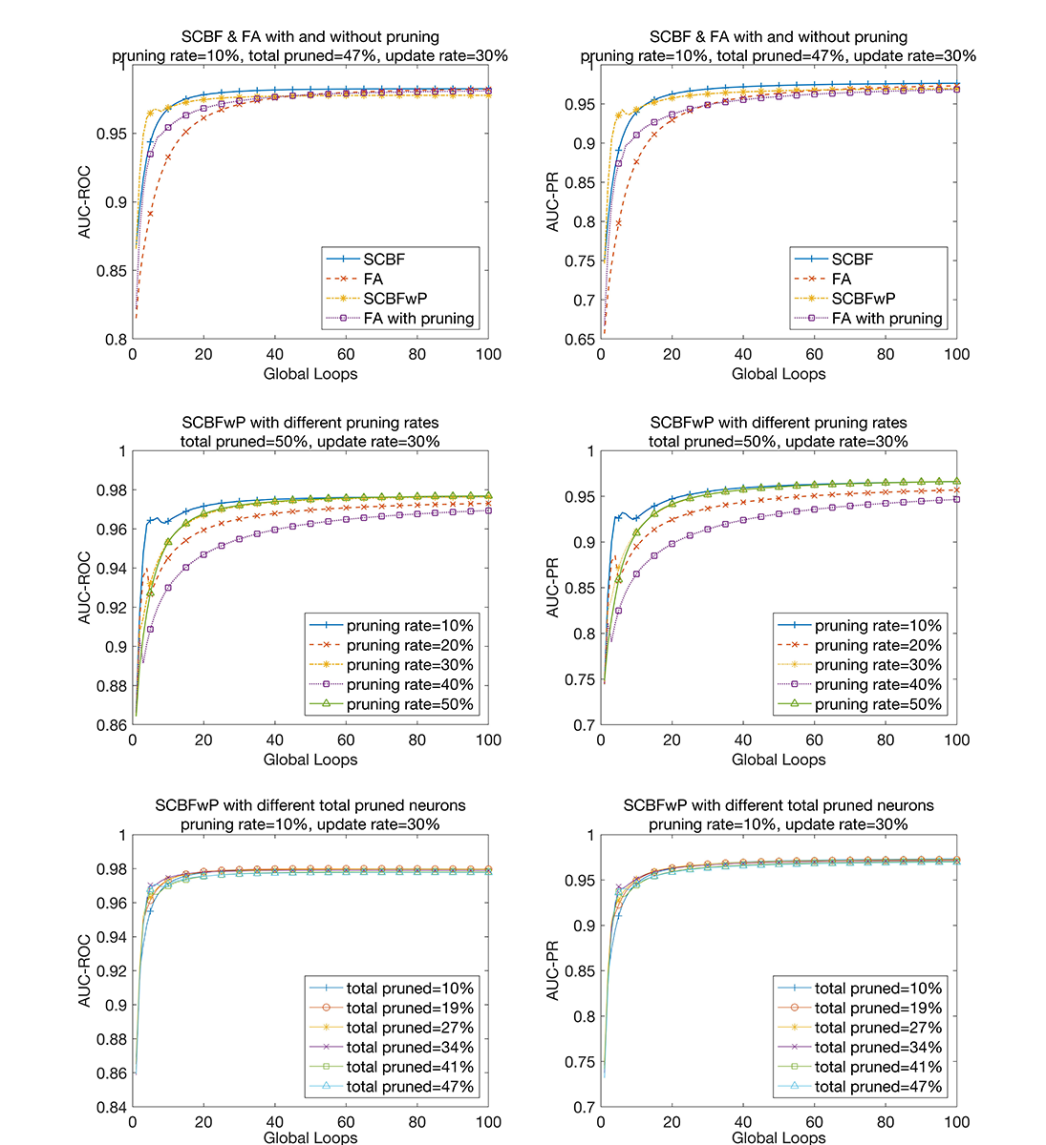
Performance of stochastic channel-based federated learning with pruning (SCBFwP). The top two graphs show the comparison between stochastic channel-based federated learning (SCBF) and federating averaging (FA) with and without pruning. The middle graphs show the performance of SCBFwP with different pruning rates. The performances of SCBFwP with different numbers of total pruned neurons are shown in the bottom two graphs. The left column shows the area under the receiver operating characteristic curve (AUC-ROC) and the right column shows the area under the precision-recall curve (AUC-PR) as performance metrics.

To speed up the training process and reduce the size of the neural network, we conducted network pruning for several loops after pretraining the model. In our trials, we set the pruning rate for each global loop to 10%, which represents the proportion of neurons to be pruned in the training loop. The total proportion of neurons to be pruned in the first several loops was set to 47%, which determines the final scale of the pruned model. Table 2 summarizes the performance of the SCBFLwP method for different update rates. Figure 7 compares the AUC-ROC and AUC-PR values of the SCBFL and federated averaging models with and without pruning.

**Table 2:** Saturated performances of stochastic channel-based federated learning with pruning with different update rates.

Update rate	AUC-ROC^a^	AUC-PR^b^
10%	0.9776	0.9695
20%	0.9772	0.9686
30%	0.9777	0.9697
40%	0.9768	0.9604
50%	0.9780	0.9695
60%	0.9774	0.9682
70%	0.9774	0.9688
80%	0.9781	0.9703
90%	0.9774	0.9676
100%	0.9775	0.9685

^a^AUC-ROC: area under the receiver operating characteristic curve.

^b^AUC-PR: area under the precision-recall curve.

As shown in Table 3, the AUC-ROC for SCBFL with pruning was reduced by 0.0048 and the AUC-PR was reduced by 0.006814. There was a reduction of 0.0012 in AUC-ROC and of 0.0047 in AUC-PR for the federated averaging method compared to federated averaging with pruning.

**Table 3:** Saturated performances of stochastic channel-based federated learning (SCBFL) and federated averaging (FA) with and without pruning.

Methods	AUC-ROC^a^	AUC-PR^b^
FA	0.9821	0.9731
SCBFL	0.9825	0.9763
FAwP^c^	0.9809	0.9683
SCBFLwP^d^	0.9776	0.9694

^a^AUC-ROC: area under the receiver operating characteristic curve.

^b^AUC-PR: area under the precision-recall curve.

^c^FAwP: federating averaging with pruning.

^d^SCBFLwP: stochastic channel-based federated learning with pruning.

Moreover, the best performance was achieved by SCBFL after 100 loops of training with an AUC-ROC of 0.9825 and an AUC-PR of 0.9763 (Table 3). The highest evaluation in the first 5 loops was obtained by the SCBFL model with pruning.

To assess the stability of our model with the pruning rate and total pruned fraction (also called the total pruned rate), we executed the models of SCBFLwP controlling the variate. First, we fixed the total pruned fraction to 50% and ran the programs with different pruning rates ranging from 10% to 50% (Table 4). As shown in Figure 7, with an increase in the pruning rate, the final performance improved and saturated more quickly under most circumstances. However, there were also exceptions with higher performances at a 10% pruning rate for both AUC-ROC and AUC-PR, and lower performances at a 40% pruning rate for AUC-PR. In the graphs in the bottom row of Figure 7, we fixed the pruning rate to 10% and executed pruning for different times ranging from 1 to 6 (Table 5). The total pruned fractions were calculated and are annotated in the corresponding labels.

**Table 4:** Saturated performances of stochastic channel-based federated learning with pruning when the total pruned proportion was fixed and the pruning rate for each training loop changed.

Pruning rate/loop	AUC-ROC^a^	AUC-PR^b^
10%	0.9765	0.9661
20%	0.9730	0.9568
30%	0.9763	0.9662
40%	0.9693	0.9465
50%	0.9769	0.9663

^a^AUC-ROC: area under the receiver operating characteristic curve.

^b^AUC-PR: area under the precision-recall curve.

**Table 5:** Saturated performances of stochastic channel-based federated learning with pruning when the pruning rate for each training loop was fixed and the total pruned proportion changed.

Total pruned proportion	AUC-ROC^a^	AUC-PR^b^
10%	0.9769	0.9731
19%	0.9797	0.9722
27%	0.9795	0.9725
34%	0.9789	0.9714
41%	0.9781	0.9703
47%	0.9778	0.9697

^a^AUC-ROC: area under the receiver operating characteristic curve.

^b^AUC-PR: area under the precision-recall curve.

As shown in Figure 6, the SCBFLwP could save 85% of the trans-information compared to federated averaging. For SCBFL, when the upload rate for channels was set to 30%, 45% of the parameters were uploaded to the server using positive selection.

### Running Time

SCBFL preserves the privacy of data by adding a channel-based upload algorithm, which will lead to an increased burden of calculations when applied to a complex neural network. However, this problem could be addressed by introducing a pruning process in several global loops. To illustrate this, the times consumed by SCBFL and federated averaging before and after pruning described in the last section are compared in Table 6. The pruning process could reduce 57% of the time for SCBFL and 48% of the time consumed by federated averaging.

**Table 6:** Time consumed by stochastic channel-based federated learning (SCBFL) and federated averaging with and without pruning.

Methods	Time (seconds)
Federated averaging	8679
Federated averaging with pruning	4508
SCBFL	19,696
SCBFL with pruning	8469

Table 7 shows that models with lower update rates tended to consume less time than those with larger update rates, indicating that choosing a lower rate for the update could better preserve the privacy as well as save time.

**Table 7:** Time consumed by stochastic channel-based federated learning with pruning with different update rates.

Update rate	Time (seconds)
10%	8339
20%	8545
30%	8469
40%	7987
50%	8359
60%	12,577
70%	9278
80%	11,462
90%	13,169
100%	13,030

Table 8 shows that different pruning rates for each global loop can equally save time. In addition, the model will consume more time if the number of pruned neurons is too small due to the executing time of the pruning process. With a fixed pruning rate, the time consumed by the model tended to decrease by reducing the model size.

**Table 8:** Time consumed by stochastic channel-based federated learning with pruning using different pruning rates for each loop or different total pruned proportions.

Pruning parameter		Time (seconds)
**Pruning rate/loop**		
	10%	11,144
	20%	8561
	30%	11,852
	40%	8389
	50%	12,000
**Total pruned**		
	10%	25,755
	19%	22,717
	27%	17,579
	34%	15,909
	41%	8050
	47%	8469

## Discussion

### Principal Results

The proposed SCBFL method computes the norms of channels in gradients resulting from the local training output after each global loop, calculates the α-percentile of the channel norms, and then sifts out the channels that have greater variation in the gradients compared with the percentile for the server update. In this method, the server seizes the information from the uploading channels with the biggest variation, achieving comparative performance to the state-of-the-art method (federated averaging), which has to convey the entire local weights to the server when updating. Figure 1 shows the relationship between the server and clients, and demonstrates the process of the server update. This confirms the intuition behind SCBFL: the importance of a feature differs when training on different datasets, and thus important information can be extracted from the channels through which features with the greatest variation pass. We could infer that less than 10% of the channels contain the most fundamental information and that ignoring the remaining information does little harm to the learning of models.

It is important to train a small-scaled deep-learning model with high processing speed. The results showed that network pruning could speed up the training process and accelerate convergence while maintaining higher performance. As expected, pruning 47% of the neurons from the network decreased the final performance due to the simplified model structure. The reduction in performance is negligible in many application situations but the acceleration in both saturating and training speed is quite beneficial, as discussed further below. Overall, these results demonstrate that SCBFL is a reliable choice for federated learning, and that the SCBFLwP method might be a better choice when a quicker saturating speed is desired.

The graphs in the first row of Figure 7 show an obvious decline in the performance of SCBFLwP, which indicates an overpruned phenomenon for our trials. This indicates a tradeoff between time efficiency and the final accuracy. However, by tuning the pruning rate for each global loop and the total pruned rate of the model, we could achieve better performance. This is because if only the redundant neurons are pruned, the model could promote its learning efficiency without retaining useless information.

Figure 7 also shows that the performance of SCBFL improved when the times of pruning were reduced. The results with a fixed pruning rate were more stable than those with a fixed total pruned rate, indicating that more attention should be paid to the selection of the pruning rate for each step when building models, and it is stable for a SCBFL model to adjust the times of neural network pruning. Therefore, after choosing a suitable pruning rate, we could appropriately increase the loops in which the model was pruned to shorten the execution time with little effect on the final performance.

### Differential Privacy Preservation

Differential privacy [23,43-46], as a strong criterion for privacy-preserving, is defined when the probability of a given output does not primarily depend on the involvement of a data point in the inputs [19]. This is useful because conventional deep learning has raised substantial privacy concerns, which may prevent a company from collecting data for model training. A model-inversion attack may extract parts of the training data through a deep-learning network, as demonstrated by Fredrikson et al [47]. One might attempt to reduce the risk of privacy leakage by adding noise to the parameters that result from the training process. However, it is hard to achieve a balance between performance and privacy preservation since stronger noise offers protection for privacy as well as worse performance. Therefore, we have been seeking methods that can help to preserve local privacy during the training process.

To address this issue, the SCBFL method realizes the function of differential privacy preservation by protecting the two sources of potential privacy leakages from federated learning: the actual values of uploaded gradients from the local participants and the mechanism by which these gradients are chosen [19]. By setting a threshold to select the parameters of gradients channel-wise, the actual values uploaded to the server are stored in a sparse tensor that is processed from SGD, a stochastic training process that has already been used for many privacy-preserving cases [48,49]. In addition, the participant could independently choose the update rate for their models, thus making it hard to track the selection of the channels used for the update, especially when they are trained individually using different datasets.

### Limitations

We proposed an update algorithm that plays a vital role in SCBFL. This algorithm involves calculation of the α-percentile of the norms and searching for channels with greater variation in the gradients than the percentile. Given the massive size of input features, the model structure has to be extended to reach high performance. However, as is the case for most deep-learning models with complex structures, the time complexity will increase with the expansion of model size. Although the neural network pruning method has been introduced to reduce the executing time, the performance of the model will slightly decrease because of the simplified model structure. Moreover, differential privacy could be further conducted with our models to evaluate the privacy-preserving ability quantitatively.

### Comparison With Prior Work

A large and representative dataset is usually required to train a neural network model. The dataset may contain sensitive information and the models should not expose the private information. Conventional methods that rely on a centrally trained model have a higher risk of privacy leakage. In conventional deep learning, the owners of the data cannot control the learning objective and have no idea of what can be inferred from their data. The federated averaging method represented progress in this regard by using iterative model averaging. Nevertheless, this approach still involves the exposure of all model parameters. The proposed SCBFL method, which improves server performance by uploading only a proportion of gradients, could address both direct and indirect privacy leakage concerns. In addition, an inverse-model attack, which extracts information from the uploaded parameters, could be hindered by the stochastic nature of our upload algorithm taking advantage of SGD. We found that even with only 10% of the channels uploaded to the server, the SCBFL model achieved an AUC-ROC of 0.9776 and an AUC-PR of 0.9695, outperforming the models that share all parameters with the server. As shown in our trials, after 100 global loops, the AUC-ROC and AUC-PR of SCBFL was 0.0033 and 0.0032 higher than that of federated averaging, respectively. Therefore, we could conclude that our method achieves comparative performance to the federated averaging method but with a higher saturating speed.

### Conclusions

We proposed a privacy-preserving approach for distributed systems whose models are trained based on any type of neural network. Our methodology involved development of a channel-based update algorithm for the server, enabling the system to achieve state-of-the-art performance without forcing the participants to reveal their inputs or model weights to the server. Addressing both direct and indirect privacy leakage concerns, our model uploads a fraction of channels in the gradients from local models to the server and could achieve better performance with only 10% of the channels uploaded, thereby reducing the redundancy of gradients while preserving privacy. Inverse-model attack, which analyzes information from the uploaded parameters, could be obstructed by the stochastic nature of our upload algorithm taking advantage of SGD. Moreover, we introduced a neural pruning process to the model, which could accelerate the training process and saturating speed with little sacrifice to the final performance. Experimental validation showed that neural network pruning could efficiently speed up the training process as well as the saturation of performance. Moreover, better performance was achieved when tuning the pruning proportion to cut off the redundant neurons in several training loops.

## Abbreviations

APoZ: average percentage of zeros
AUC-PR: area under the precision-recall curve
AUC-ROC: area under the receiver operating characteristic curve
FN: false negative
FP: false positive
FPR: false positive rate
ReLU: rectified linear unit
SCBFL: stochastic channel-based federated learning
SCBFLwP: stochastic channel-based federated learning with pruning
SGD: stochastic gradient descent
TN: true negative
TP: true positive
TPR: true positive rate
